# Polysubstance Use among Maryland High School Students: Variations across County-Level School Districts

**DOI:** 10.3390/ijerph21050639

**Published:** 2024-05-17

**Authors:** Lindsey Webb, Kechna Cadet, Rashelle Musci, Shaheen Kurani, Laura K. Clary, Danielle German, Renee M. Johnson

**Affiliations:** 1 Department of Mental Health, Johns Hopkins Bloomberg School of Public Health, Baltimore, MD 21205, USA; 2 Department of Epidemiology, Columbia University Mailman School of Public Health, New York, NY 10032, USA; 3 Health Analytics and Innovation, Delta, Hapeville, GA 30354, USA; 4 Department of Health, Behavior and Society, Johns Hopkins Bloomberg School of Public Health, Baltimore, MD 21205, USA

**Keywords:** polysubstance use, adolescence, prevention, county, school districts

## Abstract

Background: Polysubstance use is a highly prevalent public health issue, particularly among adolescents, and decisions on prevention programming and policies are often made at the local level. While there is a growing literature examining patterns of polysubstance use among adolescents, little is known about differences in those patterns across geographic regions. Methods: Using a large, representative sample of high school students from the state of Maryland (*n* = 41,091) from the 2018 Maryland Youth Risk Behavior Survey, we conducted a latent class analysis (LCA) of adolescent substance use along nine binary indicators, including past 30-day combustible tobacco, e-cigarette, alcohol, and cannabis use, as well as lifetime use of prescription opioids, cocaine, heroin, methamphetamine, and injection drug use. Measurement invariance across counties was examined using the Multiple Indicators and Multiple Causes (MIMIC) procedure. Results: The results of the LCA show three classes of adolescent substance use for the total sample: (1) low substance use, (2) commonly used substances (i.e., e-cigarette, alcohol, and cannabis use), and (3) polysubstance use. The results from the MIMIC procedure demonstrated geographic differences in students’ endorsement of specific indicators and their class membership. Conclusions: These differences demonstrate the need for an examination of local trends in adolescent polysubstance use to inform multi-tiered prevention programming and policy.

## 1. Introduction

Among adolescents who use substances, polysubstance use is very common. As just one example, one study showed that 93% of high school students in the U.S. who report past 30-day e-cigarette use also report other substance use, including alcohol, cannabis, and combustible tobacco [1]. Risk for health and psychosocial problems is higher among these youth, particularly when their use is more frequent and in larger amounts [2,3]. While there is a growing body of research characterizing patterns of adolescent polysubstance use and differences across demographic factors, there has been very little focus on differences in polysubstance use across geographic settings. However, geographic settings play a large role in shaping social environments that affect health behaviors, including substance use [4]. Enhancing knowledge about geographic variation in adolescent substance use could improve our understanding of the influence of social contexts. Moreover, prevention interventions to address adolescent substance use are often determined at the county or school district level; thus, exploring differences in substance use across counties can directly inform local prevention efforts. In this study, we use person-centered statistical techniques to investigate how patterns of adolescent polysubstance use vary across all counties within the state of Maryland.

### 1.1. Polysubstance Use in Adolescence

A main methodological challenge to characterizing polysubstance use among youth is the presence of an adequately large sample to identify all possible types and combinations of substances, as there will inevitably be a small number of adolescents who report using some drug combinations [5]. To address this challenge, researchers use latent class analysis (LCA), as it allows for subgroup identification of even low-use substance combinations. Two systematic reviews summarize the results of studies that use LCA to characterize adolescent polysubstance use, and both conclude that (1) most adolescents fall into a class characterized by abstinence or limited substance use, (2) a minority of adolescents are in a class marked by high-risk use of multiple drugs, and (3) the remainder fall into intermediary classes marked by use of the most commonly used substances, i.e., alcohol, nicotine/tobacco, and cannabis [5,6]. However, gaps in this body of literature remain. First, most studies include a limited number of substance use indicators: usually alcohol, cannabis, and tobacco. Studies that do include illicit drug use, such as cocaine and heroin, usually collapse these responses into a single category, which masks the differential use of these drugs. Second, few studies include different forms of nicotine as indicators, which prevents distinctions between youth who use combustible tobacco (e.g., cigarette use) versus vaporized nicotine (e.g., e-cigarette use). Finally, non-medical prescription opioid (NMPO) use has been largely absent as an indicator in LCA studies of adolescent substance use. The current study addresses these gaps by using an LCA approach that allows for the inclusion of multiple illicit substances, e-cigarettes, and NMPO use.

### 1.2. Variations in Patterns of Polysubstance Use

It is critical that geographic differences are examined in substance use, especially when informing policy implementation and practice at the state and local level. Adolescents in the U.S. do not demonstrate a monolith of behavior; by focusing only on national trends, researchers can miss important differences in adolescent substance use across regions. For instance, adolescent NMPO use at the national level has increased over the past two decades [7]. However, it is likely that many smaller geographic areas, such as a city, county, or school district, have low opioid use among its adolescent population; thus, focusing on reducing opioids given the national trends may not be an effective use of local resources. Instead, local efforts should be focused on implementing or expanding prevention strategies that are tailored to the specific patterns of substance use in each region.

In addition to a lack of understanding of localized trends in adolescent substance use, examining associations between polysubstance use and key demographics (e.g., sex, age, sexual and/or gender minority (SGM) status, race, and ethnicity) can further aid the tailoring of local-level prevention efforts. Although studies have demonstrated that boys [6,8,9] and older adolescents [5,6,10,11,12] have a higher likelihood of high-risk polysubstance use, there has been little exploration of other demographic factors within the literature using LCA to examine polysubstance use. For instance, SGM status is not explored in the majority of LCA studies of adolescent polysubstance use; however, those studies do suggest that SGM adolescents are more likely to be in high polysubstance use classes [3,13,14]. The associations between race, ethnicity, and classes of polysubstance use are less clear than for age, sex, and SGM status [5,6]. In studies that report race and ethnicity differences in polysubstance use class membership, the more common pattern is that Black, Latinx, and Asian youth are less likely to be in the high-risk polysubstance use classes than White youth. However, some studies do not show any race or ethnicity differences in class membership [5,6]. Given the variability in patterns of adolescent substance use across demographic factors, the current study accounts for these differences when examining variables in polysubstance use across county-level school districts.

### 1.3. Current Study

The purpose of this study is to determine heterogeneity in patterns of adolescent polysubstance use across county-based school districts in Maryland. Specifically, this study aims to identify classes of substance use among Maryland high school students, and examine county-level differences in those classes, or the extent to which the latent class structure is the same across all counties. The current study builds on previous work by (1) including an expanded set of substances as indicators, (2) exploring age, race, ethnicity, sex, and SGM status in association with latent class membership, and (3) accounting for potential measurement non-invariance at the county level to avoid bias in examining county-level differences in adolescent polysubstance use.

## 2. Materials and Methods

The current study uses data from the 2018 Maryland Youth Risk Behavior Survey/Youth Tobacco Survey (YRBS/YTS; *n* = 14,091). The biennial survey is conducted by the Centers for Disease Control and Prevention (CDC), is representative of high school students in the state, and is powered to generate prevalence estimates at the county level for the 24 county-level school districts in the state of Maryland. A two-stage cluster design was used at each county-level public school system, sampling at the school level and then at the classroom level within schools [15]; sample weights were calculated to account for the study design. Analyses were computed at the county level because, according to the Maryland Department of Legislative Services, counties are the primary unit of local government in this state and are primarily responsible for funding of basic services, including public schools and health [16].

### 2.1. Measurement of Study Variables

Students reported their past 30-day frequency of using cigarettes, cigars/cigarillos, e-cigarettes, and alcohol. For example, students were asked, “During the past 30 days, on how many days did you smoke cigarettes?”, with response options representing days of use (i.e., 0, 1 or 2, 3–5, 6–9, 10–19, 20–29, or all 30 days). Due to low prevalence, responses to the cigarette and cigar/cigarillo items were collapsed into a single variable measuring past 30-day combustible tobacco use. Students also reported on their past 30-day use of cannabis, with response options representing frequency of use over the past 30 days (i.e., 0, 1–2, 3–9, 10–19, 20–39, or 40 or more times). Students were also asked to report on their lifetime use of cocaine, heroin, methamphetamine, and NMPO use. For example, students were asked, “During your life, how many times have you taken prescription pain medication without a doctor’s prescription or differently than how a doctor told you to use it?”, with response options of 0, 1–2, 3–9, 10–19, 20–39, or 40 or more times. Injection drug use (IDU) was measured using the following item: “During your life, how many times have you used a needle to inject any illegal drug into your body?”, with response options of 0, 1, or 2 or more times. We converted all responses to binary scales to measure any past 30-day use of combustible tobacco, e-cigarettes, alcohol, and cannabis, and any lifetime use of cocaine, heroin, methamphetamine, IDU, and NMPO.

Demographic characteristics, including age (≤14, 15, 16, or ≥17 years), race, and Hispanic ethnicity (Non-Hispanic White, Non-Hispanic Black, Non-Hispanic Other Race, and Hispanic/Latinx), sex (0 = male, 1 = female), and SGM status, were included in the study. SGM status was based on items assessing sexual orientation and gender identity. To assess sexual orientation, students reported whether they identified as heterosexual/straight, gay or lesbian, bisexual, or not sure. To assess gender identity, students were asked, “Some people describe themselves as transgender when their sex at birth does not match the way they think or feel about their gender. Are you transgender?” The four response options were: no, yes, not sure, and “I don’t know what the question is asking”. A binary SGM variable was calculated with youth who indicated they were gay, lesbian, bisexual, questioning, and/or transgender being coded as SGM (1); all others were coded as not SGM (0).

### 2.2. Statistical Analyses

The current study followed recent guidance on latent variable modeling research [17,18], and used the Multiple Indicators and Multiple Causes (MIMIC) approach to test for county effects both on substance use indicators and on class membership after accounting for measurement non-invariance. The MIMIC procedure is a method for identifying differential item functioning (DIF), a source of measurement for non-invariance. DIF within a latent variable model exists when one or more indicators vary based on a predictor variable—county in this case—across one more classes. DIF is classified as uniform or nonuniform based on whether responses vary across classes. Uniform DIF exists when differences in response to indicators across levels of the predictor variable vary in a similar way across latent classes, whereas nonuniform DIF exists when differences in indicator based on the predictor variables are not the same across latent classes [17]. Using the MIMIC approach allows for the following tests: (1) county effects on substance use indicators (i.e., uniform and nonuniform DIF), and (2) county effects on class membership after accounting for DIF. The analyses included four steps: (1) conducting an LCA to determine the optimal number of classes based on the nine substance use indicators; (2) conducting a latent regression model of the associations between class membership and counties, accounting for demographic covariates, but without accounting for any potential county-level measurement non-invariance (i.e., DIF); (3) evaluating measurement non-invariance across the 24 counties using the MIMIC approach [17]; and (4) calculating a final latent regression model examining differences in class membership across the 24 counties, including demographic covariates and county-level DIF. Details regarding these four steps are summarized in Table 1. All analyses were conducted in Mplus Version 8 [19] and were conducted between September 2020 and August 2021.

## 3. Results

### 3.1. Sample Description

Demographic characteristics and substance use prevalence among the 2018 YRBS/YTS sample of Maryland high school students are displayed in Table 2. The combined, weighted sample (*n* = 257,085) has a balanced distribution of boys to girls. Forty-two percent of the students were non-Hispanic White, 35.7% were non-Hispanic Black, 9.7% were Hispanic/Latinx (any race), and the remaining 12.5% represented students who were non-Hispanic multiracial or in other racial groups (i.e., American Indian or Alaska Native, Asian, or Native Hawaiian or Other Pacific Islander). Among the 18% who identified as SGM, 84.6% identified as cisgender and sexual minority only (i.e., gay or lesbian, bisexual, or questioning), 4.3% identified heterosexual and transgender, and 11.1% identified as both a sexual and gender minority. When examining substance use, the highest prevalence estimates in the overall sample were past 30-day alcohol use (24%), past 30-day e-cigarette use (23%), past 30-day cannabis use (18%), and any lifetime NMPO use (15%). Supplemental Table S1 provides the weighted prevalence across frequency responses in the population.

### 3.2. Latent Class Analysis

**Figure 1:**
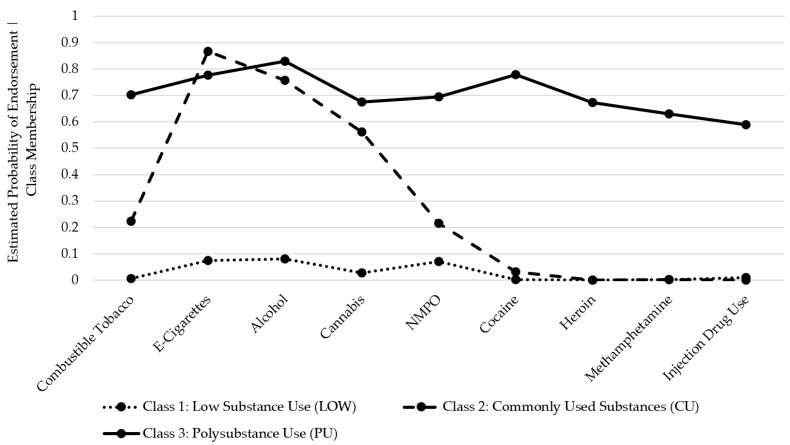
Model-estimated latent classes based on 3-class solution. Note: NMPO = non-medical prescription opioid use.

A three-class solution was selected based on the fit statistics and the substantive interpretation of the classes (see Table S2). Fit indices were compared across models with differing numbers of classes. The Akaike Information Criteria (AIC), Bayesian information Criteria (BIC) log-likelihood, and entropy all indicated that the three-class solution best fit the data, and all classes contained at least 5% of the sample, indicating stability within the model [20]. We named the classes as follows: low substance use (LOW), commonly used substance use (CU), and polysubstance use (PU; see Figure 1). The LOW class (71.6% of the population) was characterized by little to no substance use, whereas the CU class (23.1% of the population) had high estimated probabilities of e-cigarette, alcohol, and cannabis use, with a low use of other substances. The PU class contained 5.3% of the population and was characterized by reported use of a greater number of substances, including NMPO, cocaine, heroin, methamphetamine, and IDU.

### 3.3. Latent Regression Ignoring DIF

Once the optimal number of classes was identified, we then conducted a latent regression model to examine the associations between class membership and counties, while accounting for age, sex, race and ethnicity, and SGM status (Step 2). Statistically significant differences were observed in class membership, and also in age, sex, race and ethnicity, and SGM status (see Table 3). For example, when comparing the polysubstance use class to the low substance use class within the Step 2 model, the polysubstance use class had significantly more girls (OR = 3.14, *p* < 0.01) than the low substance use class. Moreover, county 1 had significantly fewer students in the polysubstance use class than county 10 (OR = 0.46, *p* < 0.01).

### 3.4. MIMIC Procedures

DIF analyses were conducted across counties to test for measurement non-invariance (see Table S3). The no-DIF model was rejected in the omnibus test (*χ*^2^ = 2,456.76, *df* = 621, *p* < 0.0001). Thus, we concluded that there was DIF associated with county for at least one indicator and proceeded with iterative testing of DIF with each substance. In those models, potential non-uniform DIF was seen in the combustible tobacco, e-cigarette, alcohol, cannabis, NMPO, cocaine, and IDU indicators. After iterative testing for uniform DIF, we created a model that had nonuniform county-level DIF on the combustible tobacco, e-cigarette, alcohol, cannabis, and NMPO items, and uniform county-level DIF on the cocaine and IDU items (M5.0 in Table S3). When compared to the model that included non-uniform DIF for the seven items (M3.0 in Table S3), M5.0 did not fit significantly worse (*χ*^2^ = 114.99, *df* = 92, *p* = 0.053).

**Figure 2:**
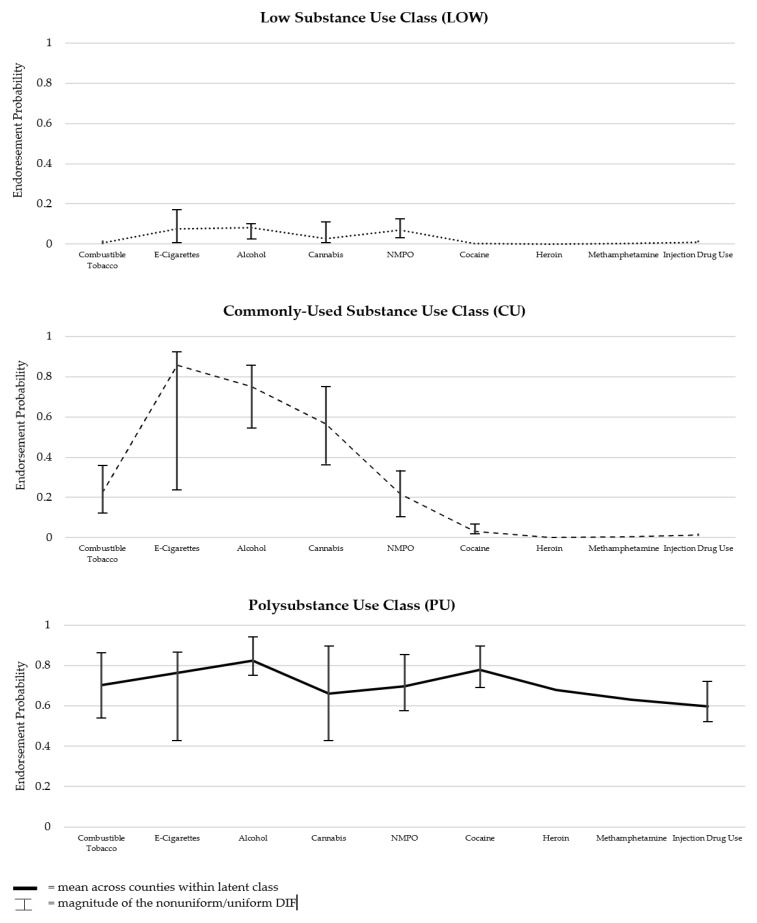
Model-estimated latent class profiles based on 3-class multiple indicator multiple cause (MIMIC) LCA with county as source of DIF. Note: Interval bars are not included for heroin or methamphetamine as no significant DIF was identified for these indicators through the MIMIC modeling process. NMPO = non-medical prescription opioid use.

Figure 2 displays each of the latent classes for the overall sample, as well as vertical bars to demonstrate the impact of DIF on class interpretation across the 24 counties (procedure 6 in Table 1). Although there were significant impacts of the county dummy variables on substance use within each class (i.e., counties showed significant DIF), the substantive interpretation of the class structure did not change. In other words, while endorsement of specific indicators differed across the 24 counties, the general structure and interpretation of the three classes were the same across the counties. Thus, we proceeded with testing the association between county and latent class membership while accounting for the effects of DIF. The null model (M7.0 in Table S3) fixed all county dummy variables to zero, while the alternative model (M7.1 in Table S3) freely estimated the county dummy variables. After accounting for DIF, county was found to still be significantly associated with class membership (*χ*^2^ = 525.24, *df* = 46, *p* < 0.0001). Thus, while the structure of the classes was similar across counties, the size of the classes differed significantly across county.

### 3.5. Final Model Accounting for DIF and Covariates

A final model was conducted including the freely estimated county variables, as well as sex, age, race and ethnicity, and SGM status as covariates. In the final model, county was found to be significantly associated with latent class membership while accounting for covariates. Moreover, when the final model was compared to the model from Step 2 that ignored DIF, there were differences in interpretation and county-level effects on class membership (see Table 3). For example, in the model ignoring DIF, there were significantly fewer students from county 15 in the CU class than the PU class compared to county 10 (OR = 0.92, *p* < 0.01). However, in the model that accounted for county-level DIF, there were significantly more students in county 15 in the CU class than the PU class compared to county 10 (OR = 1.16, *p* < 0.01). Significant differences were observed among the covariates in the final model accounting for county-level DIF. Students in the PU class were more likely to be male and identify as SGM, while students in the CU class were more likely to be female, non-Hispanic White, and older. Also, when comparing across the two models in Table 3, we noted that ignoring county-level DIF created overestimations of age and sex differences between the classes, further highlighting the need to take county-level measurement non-invariance into account.

## 4. Discussion

The purpose of the current study was to identify distinct patterns of polysubstance use among Maryland high school students and examine whether adolescent polysubstance use differed across county-level school districts in the state. Three groups emerged in our study, with most adolescents reporting low substance use, followed by commonly used substances (e.g., e-cigarettes, alcohol, and cannabis), and a small percentage reporting use of a broader array of substances (i.e., polysubstance use). This aligns with existing literature showing that most adolescents fall into the limited or low use category, with then next common pattern being alcohol, e-cigarette, and cannabis use [5,6]. Of note, among adolescents who used commonly used substances, NMPO use was relatively high. This finding may be related to the higher prevalence of NMPO use among adolescents overall (e.g., the current study shows a prevalence of approximately 15% in the Maryland high school student population). Moreover, adolescents with high polysubstance use highly endorsed all of the substance use items. The current study examined a wider range of illicit drugs than prior studies and showed that adolescents with high polysubstance use reported using these specific illicit substances, including methamphetamine, cocaine, heroin, and injection drug use. Given the severity of the impacts of illicit substance use in adolescence, prevention efforts should investigate the salient social and contextual influences on adolescents engaged in high polysubstance use and increase access to secondary interventions and treatment.

To address whether the patterns of adolescent polysubstance use differed across counties, we first examined whether counties were a potential source of DIF through the use of MIMIC models. The results of this testing show that adolescent use of specific substances differed across counties. Although it is assumed that two students within the same class would have the same responses across indicators, findings from this study indicate that two students in different counties may show differences in their use of specific substances, despite both of them being members of the same substance use class.

Overall, results from the current study show that a student’s location (i.e., county-level school district) was associated with their pattern of substance use, even after accounting for differential item functioning. This geographic variability suggests that there are important opportunities to facilitate county-level solutions to address the substance use of adolescents within their local jurisdictions. Examining how adolescent drug use patterns may vary within states and the relative influence of differing social, drug, policy, program, or other contextual factors could be invaluable to inform prevention programming and prioritization. A recent ethnographic study examined drug use patterns and experiences, service use, and capacity for expansion of harm reduction among adults who use drugs and stakeholders in each of Maryland’s counties. There were considerable similarities across counties regarding experiences of trauma, stigma, and challenges accessing services. However, differences in drug use patterns, as well as strengths and barriers in the local social, service, and municipal environments were seen across counties [21]. Although there is no comparable study among adolescents, this provides a foundation for further exploration of how geography may also be a key factor in understanding substance use patterns among young people.

Understanding how geography impacts adolescents’ risk for polysubstance use can also inform harm reduction prevention strategies that resonate with young people’s social context and population variations. Regarding the need for multipronged prevention approaches to NMPO use, one recommendation is to increase knowledge about how to access and use naloxone among Maryland youth [22]. Maryland now requires naloxone be available in public schools and to be administered by school personnel to students or others on campus (such as students’ family members) in the case of an opioid overdose [23]. Equipping adolescents with the skills to identify and mitigate harms associated with polysubstance use through indicated prevention, as opposed to narrowly focusing on preventing or avoiding use, may resonate with the intended youth audience.

In addition to county-level school district differences found in adolescent substance use, the findings from the current study show marked differences across demographic characteristics. Aligning with previous research [6], students who were male, older, and who identified as SGM were more likely to have high polysubstance use. Unexpectedly, the group with high polysubstance use also had a higher proportion of Black and Hispanic/Latinx students, and students of other races. Most of the studies using LCA to characterize adolescent substance use have been conducted among samples in which the large majority of respondents were White [8]. The population of Maryland high school students shows a greater level of racial and ethnic diversity, possibly explaining some of the differences seen within our findings. Findings from this study support the need to further examine polysubstance use patterns in racially and ethnically diverse samples.

Although the current study furthers work in examining adolescent polysubstance use, there are key limitations. First, measurement of substance use was limited by items included in the YRBS/YTS. Thus, precision in measuring more of the illicit substances (i.e., heroin, methamphetamine, etc.) was limited, as only lifetime use of these substances was measured. Second, the substance use items were collapsed into binary measures for the latent class analysis, which may potentially limit our understanding of differences in frequency of use. However, examination of the full items in the sample shows that most students who reported lifetime use of these substances reported using one or two times (see Table S1). Although the study was from the Maryland YRBS/YTS and powered to estimate prevalence at the county level within the state, the results may not be generalizable to adolescents at higher risk for substance use, such as adolescents who have left school, or who are homeless or incarcerated. Furthermore, future studies should consider assessing polysubstance use temporally to explore how patterns of substance use change over time [24]. The current study was not able to examine multilevel LCA models due to the relatively small number of counties and a lack of county-level covariates (e.g., availability of substances). Future studies with data from more counties should explore multilevel LCA models to examine how latent class structures may differ across geographic location and how the characteristics of counties may contribute to differences in latent classes of adolescent polysubstance use. Additionally, data from the 2018 Maryland YRBS/YTS were used within the current study. More recent data from the Maryland YRBS/YTS have since been made available and future studies should explore county-level differences given potential changes in substance use trends since the COVID-19 pandemic. Finally, the state of Maryland is led by county-level government and school districts aligned with counties, thus making counties the best area-level unit of analysis for this study based on communicability of results, relevance of substance use distribution, and utility to inform local-level prevention efforts. However, although the county-level data represent a strength in this analysis, it is important to consider that, in other states, many counties are geospatially large and heterogenous in nature, and may not align with school districts or systems that make local-level prevention programming and policy decisions. Therefore, using other granular-level geographical aggression units when assessing polysubstance use, such as small school district, ZIP code, or Census tract units, can help provide pertinent information on the local variation of polysubstance use in order to tailor interventions. Future studies should consider assessing polysubstance use across multiple scales and zonal systems to show ranges of possible distributions [25].

Despite these limitations, the current study has a multitude of strengths. First, the sample used from the YRBS/YTS is sizable and representative of high school students in the state of Maryland. Moreover, the dataset was powered to the county level, allowing for the testing of measurement invariance and county-level effects on adolescent polysubstance use. The survey also incorporated the measurement of key demographic characteristics that were included in our final model, including sex, race and ethnicity, and sexual and gender minority status. Our model also incorporated multiple illicit substances, providing critical detail of polysubstance use among adolescents.

## 5. Conclusions

The findings from the current study, which demonstrate how adolescent substance use varies by county-level school districts, may provide important information for substance use prevention programming at the local level. County-level prevention programming may use information from the current results to identify groups of high school students who may be at risk of polysubstance use based on their district and demographic characteristics. As an example, local school district leadership may determine the scope of the issue of polysubstance use within their district, as well as what specific substances may be a particular issue among students with polysubstance use (i.e., uniform and nonuniform DIF). This information can then be utilized to create stronger block grant applications to support targeted and tailored prevention efforts. Future studies in other states can use similar methods to identify distinct patterns at the local level and create tailored prevention programming and policies to address substance use among students who are at highest risk.

## Figures and Tables

**Table 1:** Description of latent class analysis, latent regression, and Multiple Indicators and Multiple Causes (MIMIC) steps and procedures.

Step	Procedure	Model *	Description
1	--	--	**Latent Class Analysis.** The number of latent classes was selected by comparing standard fit indices, including Akaike Information Criteria, Bayesian and Sample-Size Adjusted Bayesian Information Criteria, the Lo–Mendell–Rubin adjusted likelihood ratio test, the Vuong–Lo–Mendell–Rubin likelihood ratio test, and entropy.
2	--	--	**Latent Regression Ignoring DIF.** Model the associations between the county variables and latent class membership using the 1-step latent class regression approach including age, sex, race/ethnicity, and SGM status as covariates.
3	[1]	M1.0 M1.1	**Omnibus Test of DIF.** County dummy variables were examined as sources of DIF, starting with an omnibus test that compared a null model with invariance for all nine indicators regarding the county variables (i.e., no DIF model), to a model with class-specific DIF on every item regarding the county variables (i.e., full DIF model). If the null hypothesis was rejected, testing was continued. The null hypothesis is that there is no DIF for any indicator; it is rejected if there was evidence of DIF for at least one indicator.
	[2]	M2.0.1–M2.1.9	**Item-Level Testing of Non-Uniform DIF.** A series of models were conducted for each indicator to evaluate whether non-uniform DIF was present for the county variables, where direct effects of the county variables to the indicators differed by latent class—null hypothesis is no DIF for each indicator.
	[3]	M3.0	**Model with Non-Uniform DIF.** A MIMIC model with non-uniform DIF with indicators identified in procedure 2 was compared to the full DIF model to determine that it was not a statistically significantly worse fitting to the data.
	[4]	M4.1–M4.7	**Testing for Uniform DIF.** Items were then evaluated for uniform DIF, where DIF is more constrained and the effect of the county variables on the indicators is the same across the latent classes.
	[5]	M5.0	**Model with Uniform DIF.** An additional MIMIC model that included uniform DIF for indicators identified in procedure 4 is compared to the MIMIC model from procedure 3.
	[6]	--	**Evaluating Practical and Substantive Impact of DIF.** Once all sources of DIF were identified, we evaluated its practical and substantive impact. We plotted the model-estimated latent class profiles across the 24 counties to make a substantively informed determination of the comparability of the classes.
	[7]	M7.0 M7.1	**Omnibus Test of County on Latent Class Membership.** If the latent classes are still comparable, two additional models were conducted and compared. The first fixed all regression coefficients for classes on county at zero, and the second with the regression being freely estimated. If the first model fits significantly worse than the second, then there is evidence that latent class membership differs across counties.
4	--	--	**Latent Regression Accounting for DIF.** Evaluate the associations between the county variables and latent class membership using the 1-step latent class regression approach with direct effects from the county variables to the items included based on the DIF analysis, as well as age, sex, race/ethnicity, and SGM status as covariates. The final model in this procedure accounts for uniform and non-uniform DIF.

Note: DIF = differential item functioning, SGM = sexual and/or gender minority, -- = procedure and model are not relevant for steps 1, 2, and 4, and model is not relevant for procedure 6. * Model numbers align with model results listed in Table S3 in Supplementary Materials.

**Table 2:** Prevalence of demographic characteristics and substance use among Maryland high school students, 2018 (unweighted *n* = 41,091, weighted *n* = 257,085).

	Overall Sample		Range in Prevalence across Counties			
			Min		Max	
	%	95% CI	%	95% CI	%	95% CI
**Demographic Characteristics**						
Male	50.99	49.54, 52.44	48.64	42.45, 54.86	53.60	45.74, 61.28
Non-Hispanic White	42.03	40.16, 43.93	1.69	1.11, 2.57	86.44	85.15, 87.64
Non-Hispanic Black	35.72	33.31, 38.20	1.10	0.58, 2.10	85.59	80.53, 89.50
Non-Hispanic All Other	12.53	11.37, 13.79	1.78	1.46, 2.16	26.99	24.05, 30.14
Hispanic/Latino, any race	9.72	8.73, 10.80	4.53	3.77, 5.43	17.19	13.35, 21.86
Sexual and/or Gender Minority (SGM)	18.16	17.31, 19.04	13.23	11.38, 15.33	21.91	18.38, 25.92
**Substance Use Prevalence**						
Combustible tobacco, past 30-day	8.66	8.00, 9.36	5.14	3.88, 6.77	17.23	13.73, 21.41
E-Cigarettes, past 30-day	23.03	21.96, 24.12	10.65	8.12, 13.86	42.01	37.13, 47.05
Alcohol, past 30-day	24.05	22.91, 25.23	18.28	15.68, 21.19	40.38	36.62, 44.26
Cannabis, past 30-day	17.56	16.66, 18.50	13.29	11.18, 15.72	26.35	22.89, 30.13
NMPO, lifetime	14.57	13.87, 15.29	8.90	7.15, 11.04	21.00	18.18, 24.13
Cocaine, lifetime	4.83	4.33, 5.38	3.17	2.01, 4.97	13.05	9.33, 17.95
Heroin, lifetime	3.71	3.18, 4.33	1.98	0.96, 4.01	11.60	8.61, 15.46
Methamphetamine, lifetime	3.68	3.21, 4.21	1.83	0.92, 3.59	10.28	7.72, 13.56
IDU, lifetime	4.08	3.62, 4.59	2.07	1.44, 2.96	9.18	7.02, 11.93

Note: All percentages and confidence intervals are weighted. NMPO = non-medical prescription opioid use, IDU = injection drug use.

**Table 3:** Associations between class membership and counties, age, sex, race, ethnicity, and sexual and gender minority status in model accounting for DIF and model ignoring DIF.

	PU (REF) vs. LOW		PU (REF) vs. CU		LOW (REF) vs. CU	
	Step 2 Model	Step 4 Model	Step 2 Model	Step 4 Model	Step 2 Model	Step 4 Model
	OR	OR	OR	OR	OR	OR
Age						
14 years or younger	REF	REF	REF	REF	REF	REF
15 years old	1.23 **	1.22 **	2.23 **	2.19 **	1.81 **	1.80 **
16 years old	0.90	0.90	2.46 **	2.43 **	2.72 **	2.70 **
17 years or older	0.58 **	0.58 **	2.18 **	2.16 **	3.76 **	3.75 **
Sex						
Boys	REF	REF	REF	REF	REF	REF
Girls	3.14 **	3.10 **	4.24 **	4.16 **	1.35 **	1.34 **
Race and Ethnicity						
Non-Hispanic White	REF	REF	REF	REF	REF	REF
Non-Hispanic Black	0.78	0.79	0.47 **	0.49 **	0.60 **	0.62 **
Non-Hispanic Other	0.90	0.90	0.67 **	0.69 **	0.75 **	0.77 **
Hispanic/Latinx	0.38 **	0.38 **	0.35 **	0.35 **	0.91	0.91
Sexual/Gender Minority Status						
Not SGM	REF	REF	REF	REF	REF	REF
SGM	0.21 **	0.21 **	0.24 **	0.25 **	1.14 *	1.17 *
Counties ^+^						
County 1	0.46 **	0.47 **	0.59 **	0.60 **	1.27 **	1.27 **
County 2	0.60 **	0.59 **	0.52 **	0.61 **	0.88 **	1.04 **
County 3	0.78 **	0.76 **	0.56 **	0.59 **	0.71 **	0.77 **
County 4	0.68 **	0.68 **	0.80 **	0.79 **	1.19 **	1.16 **
County 5	0.60 **	0.57 **	0.83 **	0.82 **	1.39 **	1.43 **
County 6	0.87 **	0.89 **	0.79**	0.82 **	0.90 **	0.92 **
County 7	0.74 **	0.79 **	1.01	0.98 **	1.36 **	1.23 **
County 8	0.54 **	0.51 **	0.29 **	0.39 **	0.54 **	0.76 **
County 9	0.22 **	0.23 **	0.19 **	0.18 **	0.83 **	0.78 **
County 11	0.40 **	0.42 **	0.48 **	0.48 **	1.18 **	1.16 **
County 12	0.90 **	0.92 **	0.89 **	0.94 **	0.99 **	1.02 **
County 13	1.15 **	1.07 **	0.70 **	0.79 **	0.61 **	0.73 **
County 14	0.33 **	0.36 **	0.64 **	0.63 **	1.92 **	1.72 **
County 15	1.63 **	1.51 **	0.92 **	1.16 **	0.57 **	0.77 **
County 16	0.86 **	0.75 **	0.30 **	0.60 **	0.35 **	0.81 **
County 17	0.50 **	0.57 **	0.78 **	0.81 **	1.56 **	1.41 **
County 18	0.82 **	0.91 **	0.85 **	0.94 **	1.05 **	1.03 **
County 19	0.40 **	0.38 **	0.51 **	0.72 **	1.27 **	1.90 **
County 20	0.50 **	0.47 **	0.64 **	0.72 **	1.28 **	1.52 **
County 21	0.77 **	0.78 **	0.67 **	0.71 **	0.87 **	0.91 **
County 22	0.49 **	0.51 **	0.43 **	0.49 **	0.89 **	0.96
County 23	0.51 **	0.55 **	0.66 **	0.65 **	1.30 **	1.19 **
County 24	0.37 **	0.38 **	0.21 **	0.24 **	0.55 **	0.62 **

Note: Step 2 model ignored county-level DIF. Step 4 model accounted for county-level DIF. PU = Polysubstance use class, LOW = Low substance use class, CU = Commonly used substance class. ^+^ For county dummy variables, county 10 served as the reference group. * *p* < 0.05, ** *p* < 0.01.

## Data Availability

Data from the Maryland Youth Risk Behavior Survey/Youth Tobacco Survey are available upon request at https://health.maryland.gov/phpa/ccdpc/Reports/Pages/YRBS-Main.aspx. Accessed on 25 August 2020.

## Supplementary Materials

The following supporting information can be downloaded at: https://www.mdpi.com/article/10.3390/ijerph21050639/s1, Table S1: Weighted Prevalence (%) of Maryland High School Students Who Reported Past 30-Day and Lifetime Substance Use Across Frequency Responses in 2018; Table S2: Fit Statistics for Latent Class Analysis; Table S3: Model Comparison for Stepwise DIF Testing for County with Three-Class Model of Polysubstance Use.
