# Supplementary material for: Polysubstance Use among Maryland High School Students: Variations across County-Level School Districts
Source: Int J Environ Res Public Health. 2024 May 17;21(5):639. doi: 10.3390/ijerph21050639 (PMC11121598; doi:10.3390/ijerph21050639)
Supplement: Supplementary file 1 [file ijerph-21-00639-s001.zip › ijerph-2937507-supplementary.pdf]

Table S1. Weighted prevalence (%) of Maryland high school students who reported past 30-day and lifetime substance use across frequency responses in 2018.

	Any	0 days	1 or 2 days	3 to 5 days	6 to 9 days	10 to 19 days	20 to 29 days	All 30 days	Missing
Cigarette, past 30-day	4.88	93.72	2.07	0.70	0.57	0.49	0.28	0.77	1.39
Cigar/cigarillo, past 30-day	5.89	91.83	2.55	1.19	0.88	0.40	0.26	0.61	2.29
E-Cigarette, past 30-day	20.97	70.12	7.97	3.60	2.09	2.32	1.64	3.35	8.91
Alcohol, past 30-day	22.15	69.96	12.56	4.96	2.45	1.21	0.38	0.59	7.88
	Any	0 times	1 or 2 times	3 to 9 times	10 to 19 times	20 to 39 times	40 or more times	--	Missing
Cannabis, past 30-day	16.26	76.35	6.77	4.02	1.92	1.48	2.07	--	7.39
NMPO, lifetime	13.89	81.46	6.51	3.56	1.73	0.79	1.30	--	4.65
Cocaine, lifetime	4.60	90.79	2.12	0.88	0.53	0.37	0.70	--	4.61
Heroin, lifetime	3.51	91.17	1.39	0.94	0.44	0.19	0.55	--	5.31
Methamphetamine, lifetime	3.50	91.66	1.51	0.69	0.45	0.19	0.66	--	4.84
	Any	0 times	1 time	2 or more times	--	--	--	--	Missing
IDU, lifetime	3.83	90.04	2.39	1.44	--	--	--	--	6.13

Note: NMPO = non-medical prescription opioid, IDU = injection drug use.

Table S2. Fit Statistics for Latent Class Analysis, n=41,019

	**Latent Class Models**				
**Fit statistic**	**1 class**	**2 class**	**3 class***	**4 class**	**5 class**
No. of parameters	9	19	**29**	39	49
Model maximum Log-likelihood value	-119,950.90	-99,051.31	**-92,688.93**	-92,260.68	-91,896.29
Akaike's Information Criterion (AIC)	239,919.80	198,140.63	**185,435.86**	184,599.36	183,890.57
Bayesian Information Criterion (BIC)	239,997.37	198,304.39	**185,685.82**	184,935.52	184,312.92
Sample Size Adjusted BIC	239,968.77	198,244.01	**185,593.66**	184,811.58	184,157.19
Entropy	N/A	.836	**.867**	.832	.845
Vuong-Lo-Mendel-Rubin Likelihood Ratio Test (*p*)	N/A	41,799.18 (< .0001)	**12,724.76 (< .0001)**	856.50 (< .0001)	728.80 (< .0001)
Lo-Mendel-Rubin Likelihood Ratio Test LMR-LRT (*p*)	N/A	41,409.23 (< .0001)	**12,606.05 (< .0001)**	848.51 (< .0001)	722.00 (< .0001)
Size of each of the component classes for each *c* class model					
Class 1	100%	72.8%	**71.6%**	70.4%	70.5%
Class 2	--	27.2%	**23.1%**	20.8%	20.4%
Class 3	--	--	**5.3%**	4.9%	4.0%
Class 4	--	--	--	3.9%	2.7%
Class 5	--	--	--	--	2.3%

* The 3-class model was the preferred solution based on fit statistics.

Table S3. Model Comparison for Stepwise DIF Testing for County with 3-Class Model of Polysubstance Use

Procedure	Model	Model Description		LL	npar	Comparison	LRT	df	*p*
1	M1.0	MIMIC	No DIF	-92,147.61	75	M1.0 vs. M1.1	2456.76	621	< .0001
	M1.1		All DIF	-90,919.23	696				
2	M2.0.1	Combustible Tobacco	No DIF	-37,057.19	51	M2.0.1 vs M2.1.1	146.32	69	< .0001
	M2.1.1		NU DIF	-36,984.03	120				
	M2.0.2	E-cigarettes	No DIF	-39,963.76	51	M2.0.2 vs M2.1.2	416.18	69	< .0001
	M2.1.2		NU DIF	-39,755.67	120				
	M2.0.3	Alcohol	No DIF	-42,963.42	51	M2.0.3 vs M2.1.3	194.39	69	< .0001
	M2.1.3		NU DIF	-42,866.22	120				
	M2.0.4	Cannabis	No DIF	-39,998.09	51	M2.0.4 vs M2.1.4	561.46	69	< .0001
	M2.1.4		NU DIF	-39,717.36	120				
	M2.0.5	NMPO	No DIF	-43,088.65	51	M2.0.5 vs M2.1.5	358.81	69	< .0001
	M2.1.5		NU DIF	-42,909.25	120				
	M2.0.6	Cocaine	No DIF	-32,439.71	51	M2.0.6 vs M2.1.6	103.79	69	.004
	M2.1.6		NU DIF	-32,387.82	120				
	M2.0.7	Heroin	No DIF	-31,127.39	51	M2.0.7 vs M2.1.7	50.44	69	.955
	M2.1.7		NU DIF	-31,102.17	120				
	M2.0.8	Metham-phetamine	No DIF	-31,461.97	51	M2.0.8 vs M2.1.8	52.39	69	.932
	M2.1.8		NU DIF	-31,435.77	120				
	M2.0.9	IDU	No DIF	-33,069.77	51	M2.0.9 vs M2.1.9	94.61	69	.022
	M2.1.9		NU DIF	-33,022.47	120				
3	M3.0	MIMIC	Combustible tobacco, e-cigarettes, alcohol, cannabis, NMPO, cocaine, IDU w/ NU DIF; heroin and methamphetamine w/ no DIF	-91,001.40	558	M1.0 vs M3.0	2292.42	483	< .0001
						M3.0 vs M1.1	164.34	138	.063
4	M4.1	MIMIC	Combustible tobacco w/ U DIF; all others w/ NU DIF	-91,035.82	512	M4.1 vs M3.0	68.83	46	.016
	M4.2		E-cigarettes w/ U DIF; all others w/ NU DIF	-91,087.93	512	M4.2 vs M3.0	173.06	46	< .0001
	M4.3		Alcohol w/ U DIF; all others w/ NU DIF	-91,041.39	512	M4.3 vs M3.0	79.99	46	.001
	M4.4		Cannabis w/ U DIF; all others w/ NU DIF	-91,088.93	512	M4.4 vs M3.0	175.05	46	< .0001
	M4.5		NMPO w/ U DIF; all others w/ NU DIF	-91,059.57	512	M4.5 vs M3.0	116.34	46	< .0001
	M4.6		Cocaine w/ U DIF; all others w/ NU DIF	-91,030.58	512	M4.6 vs M3.0	58.36	46	.104
	M4.7		IDU w/ U DIF; all others w/ NU DIF	-91,030.27	512	M4.7 vs M3.0	57.75	46	.115
5	M5.0	MIMIC	Combustible tobacco, e-cigarettes, alcohol, cannabis, NMPO w/ NU DIF; cocaine and IDU w/ U DIF; heroin and methamphetamine w/ no DIF	-91,058.89	466	M5.0 vs M3.0	114.99	92	.053
7	M7.0	MIMIC	Constrain class membership on county (class-county association = 0)	-91,326.51	420	M7.0 vs M7.1	535.24	46	< .0001
	M7.1		Class membership on county (freely estimated)	-91,058.89	466				

Note. LL = Model maximum log likelihood value; npar = number of free parameters; LRT = likelihood ratio test chi-square statistic; df = degrees of freedom; MIMIC = Multiple Indicator Multiple Cause; U DIF = uniform DIF; NU DIF = non-uniform DIF, NMPO = non-medical use of prescription opioids, IDU = injection drug use. Procedure 6 involves evaluating substantive impact of DIF – no statistical tests are done in this procedure, thus its exclusion from the above table.
